# Combining Statistical Tools and Ecological Assessments in the Study of Biodeterioration Patterns of Stone Temples in Angkor (Cambodia)

**DOI:** 10.1038/srep32601

**Published:** 2016-09-06

**Authors:** G. Caneva, F. Bartoli, V. Savo, Y. Futagami, G. Strona

**Affiliations:** 1 Department of Science, Roma Tre University, Viale Marconi 446, 00146 Rome, Italy; 2 Hakai Institute, Simon Fraser University, 8888 University Drive, Burnaby BC V5A 1S6, Canada; 3 National Research Institute for Cultural Properties, Tokyo, 13-43, Ueno Park, Taito-ku, Tokyo, 110-8713 Japan; 4 European Commission, Joint Research Center, Institute for Environment and Sustainability, Ispra, Italy

## Abstract

Biodeterioration is a major problem for the conservation of cultural heritage materials. We provide a new and original approach to analyzing changes in patterns of colonization (Biodeterioration patterns, BPs) by biological agents responsible for the deterioration of outdoor stone materials. Here we analyzed BPs of four Khmer temples in Angkor (Cambodia) exposed to variable environmental conditions, using qualitative ecological assessments and statistical approaches. The statistical analyses supported the findings obtained with the qualitative approach. Both approaches provided additional information not otherwise available using one single method. Our results indicate that studies on biodeterioration can benefit from integrating diverse methods so that conservation efforts might become more precise and effective.

Different biological agents can colonize stone artifacts and monuments in archaeological sites causing their biodeterioration^1^,^2^,^3^,^4^. These biological agents generally include bacteria (chemolithoautotrophic and chemoorganotrophic), cyanobacteria, algae, fungi, lichens, mosses, and even vascular plants in the final stages of colonization^5^,^6^,^7^. The BPs of the various organisms depend mostly on edaphic conditions, macro and micro-environmental factors, and frequency of maintenance^7^,^8^,^9^. The colonization of stone artifacts and monuments is also affected by the complex interactions among organisms that are co-existing on the stone or growing at different stages of colonization^8^,^10^.

Research on colonization dynamics and BPs is generally qualitative, with little attention to ecological considerations (e.g., species niche)^10^. Only a few studies, especially among those focusing on lichen communities, have attempted to understand better the relationship between community composition and environmental factors^11^,^12^. A minority of researchers have suggested using an ecological approach in assessing BPs (e.g., on the volcanic substratum in the archaeological site of Copán, Honduras^12^; on the mural paintings of the Crypt of the Universal Sin, Matera, Italy^13^). However, such studies mostly use a qualitative approach for assessing the spatial arrangement (i.e., ecotones) of colonizing communities and lack a robust statistical background that could allow for a generalization of the results. In this context, co-occurrence analysis^14^,^15^ could provide an objective way to identify non-random patterns of associations among the different communities responsible for BPs. This is because the co-occurrence analysis aims at testing if two species tend to occur in the same area more (or less) often than expected according to different ecological hypotheses^16^.

Most studies have used the co-occurrence analysis mainly to investigate the importance of ecological competition in the establishment of biological communities (e.g., ref. 17). Lately, due to the increasing interest in the study of ecological networks (e.g., ref. 18), the co-occurrence analysis has been proposed as a useful alternative to empirical studies in the identification of species interactions^19^. The general assumption behind this idea is that if two species are found together significantly more often than a random expectation, then it is reasonable to assume that they are linked by some kind of ecological relationship^20^. A systematic identification of the pairwise relationships between species in the same community generates a network of interactions among species, providing information on community structure and functioning^21^. This approach has been developed and tested mostly on species interactions *within* biological communities. Here we show how it can be successfully applied also to investigate non-random relationships *between* biological communities.

In the present work, and in the general context of biodeterioration studies, unraveling the hidden relationships within and between colonizing communities could offer new insights on how these communities function and grow. Combining the co-occurrence analysis and an ecological niche modeling^22^,^23^ can help researchers to assess how much the structure of the communities responsible for BPs is influenced by environmental factors. These analyses are valuable for conservation purposes: identifying the survival limits and ecological requirements of different colonizing species and communities may suggest preventive and/or conservative interventions against undesirable biological growths^10^,^24^,^25^.

Here we combine a qualitative assessment with various statistical approaches using temples in the archaeological site of Angkor (Cambodia) as test sites. We test the possibility of obtaining a model suitable to predict the changes occurring in BPs in relation with changes in environmental factors using an ecological analysis of the main biological communities and related BPs that are detectable in exposed stone surfaces of four temples (Ta Prohm, Ta Nei, Bayon and Ta Keo) (Fig. 1) with different microclimatic conditions. After its abandonment in the 15^th^ century the tropical forest gradually recolonized the city of Angkor and its monuments^26^. Today, the city of Angkor is one of the most important archaeological sites in Southeast Asia and a UNESCO World Heritage Site (http://whc.unesco.org/en/list/668).

## The Importance of Ecological Factors

The ecological niche of colonizing species determines the colonization dynamics. The ecological requirements that shape species niches can be seen as limiting factors, i.e., those environmental conditions and/or resources present in a quantity proximal to the upper or lower tolerance limit of an organism^27^. The combination of ecological requirements and limiting factors can determine distinctive spatial arrangements (i.e., ecotones) among communities and can affect their related BPs. In tropical wet climates, with high water and light availability, colonizing species find ideal growth conditions^25^,^28^. This is for example the case of Mayan temples in Mesoamerica^12^,^29^,^30^,^31^, or Hindu and Buddhist temples in South-Asia, such as the Khmer temples of Angkor.

The communities responsible for biological colonization on specific substrata tend to be similar among sites with similar environmental/climatic conditions. In fact, cyanobacteria, algae, and lichens, and sometimes also mosses and vascular plants form colonizing communities that tend to dominate on exposed stones in favorable climates, with changes in species that depend on the surrounding environmental factors. Previous research has shown that BPs of photoautotrophic communities vary mostly in relation to shadowing by forest canopy, which in turn might affect water availability (humidity and precipitation) and solar radiation, but can vary also in relation to exposure and inclination of stone surfaces^32^. The influence of other factors, such as organic nutrients and minerals, which depend on the substratum but also potentially on eutrophication or air pollution, becomes stronger only when the previously cited factors reach threshold limits for specific photoautotrophic communities^10^. Conversely, fungi and other heterotrophic organisms, depending less strictly on water and solar radiation, become prevalent in the colonization of artifacts in polluted areas and/or in conditions of eutrophication^33^, and in extreme environmental conditions^34^.

## Results

In the analyzed temples in Angkor, the distribution of various colonizing communities is strongly affected by light and water availability (Table 1). In the more than 200 field observations carried out on the temples, we observed the recurrence of only eight communities and related BPs. Field observation highlighted the recurrence of only 16 possible transitions among the 28 theoretical possible combinations of BPs 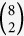. These 16 transitions, observed in the field in different conditions of shadowing by the surrounding forest, are shown in Fig. 2A. The ecological space of the colonizing communities along with their ecotone transitions related to the principal limiting ecological factors are showed in Fig. 2B.

We found a high agreement between the field identification of transitions and the results of the co-occurrence analysis, with some minor differences. In particular, the co-occurrence analysis (Table 2) identified 16 significant transitions, which included most (13 out of 16) of the transitions observed in the field, plus three additional transitions not observed. We also found three associations of BPs that were detected in the field, but not by the co-occurrence analysis. These are the transition between Cyanobacteria (*Scytonema* and *Gloeocapsa*) and the lichen community with *Pyxine* (B-E), which resulted in the co-occurrence analysis as marginally significant (p value ~ 0.06). Also the transitions between the two lichen communities of *Pyxine*-*Cryptothecia* (E-F) and the *Lepraria* lichen-mosses (D-G) both showed a non-significant p value. The identified associations among communities were then used to build a weighted network of interactions that provided an effective way to visualize the ecological relationships between transitions (Fig. 2C).

Our data showed that, for the temples in Angkor, light and water are the most critical factors in explaining the distribution of the colonizing communities. In particular, we found that the combination among these environmental factors had a very high explanatory power, having an ‘overall score’ (*SC*) (see also ref. 35) value of 0.46. However, our data also indicated that these two factors, when considered separately, explained poorly the distribution of the associations, with light (*SC* = 0.14) being slightly more informative than water (SC = 0.10).

### Integrating qualitative assessments with statistical approaches

The statistical analyses supported the findings obtained with the qualitative assessment in the field. For instance, the fact that the two transitions (D-G and E-F) had non-significant p values (Table 2) is coherent with the ecological qualitative approach showed in Fig. 2B. These transitions occurred only in particular conditions where factors other than light and water were involved in determining the association of these two communities. In the case of the D-G transition, the result in a non-significant value might be due to the fact that D-G transitions can be found only in presence of high humidity (such as conditions of rising damp or water percolation). As regards the lichen communities dominated by *Pyxine* sp. and *Cryptothecia* sp. (E-F), we noted in the field that they often showed a certain overlapping due to their similar ecological spaces (niches) and this might have altered the analysis of their co-occurrence.

The consistency between the transitions identified in the field and those identified by the co-occurrence analysis indicates that both the ecological qualitative assessments and the co-occurrence analysis can provide robust, complementary results. This is because even if some transitions were not observed in the field, they appear to be ecologically possible, and probably correlated to changes in additional ecological factors. For instance, some of the additional transitions determined by the co-occurrence analysis could also indicate some unusual ecological conditions, as in the case of the transition between the cyanobacteria community with *Endocarpon* and the lichen community of *Lepraria* (C-D) and possibly also other two transitions in Table 2 [*Trentepohlia* - *Cryptothecia* (A-F); *Trentepohlia*- *Pyxine* (A-E); see also Fig. 2B]. In this sense, the co-occurrence approach can represent a powerful tool to assess the potential growth of transitions that were not observed, but could appear once additional factors (change in edaphic factors, inclination, or nutrients) start to play a significant ecological role.

## Discussion

The statistical analyses supported some of the findings of the qualitative assessment but also provided some additional complementary information. Our results indicated that biodeterioration studies can benefit from integrating ecological qualitative observations with quantitative ecology analytical techniques. This is true whether we consider simple presence-absence data of colonizing communities or the evaluation of ecological niches of colonizing species.

Our results indicated that the spatial distribution of colonizing communities and the transitions among them are significantly affected by light and water availability. However, considering the wide range of ecological factors that could affect BPs, it would be worth exploring further ecological niche modeling techniques. This could help defining BPs at a larger scale of bioclimatic regions, i.e., by combining high-resolution climatic data and other environmental and ecological factors, creating a wider model for each bioclimatic area related to different stone materials. This research could be valuable for optimizing conservation efforts by identifying conditions with high susceptibility to BPs.

The proposed ecological scheme can be a useful tool for the conservation of monuments. This scheme could allow predicting the changes of BPs when ecological factors change. Furthermore, it represents a useful tool for evaluating the possible use of indirect methods for biodeterioration control (i.e., planning interventions that would change the environmental conditions that affect BPs). In this way, it would be possible to fine-tune interventions creating more suitable or suboptimal microclimatic conditions for stone conservation, balancing between conditions that would cause chemical weathering and biodeterioration phenomena.

## Methods

### Site selection and ecological features

**Figure 1:**
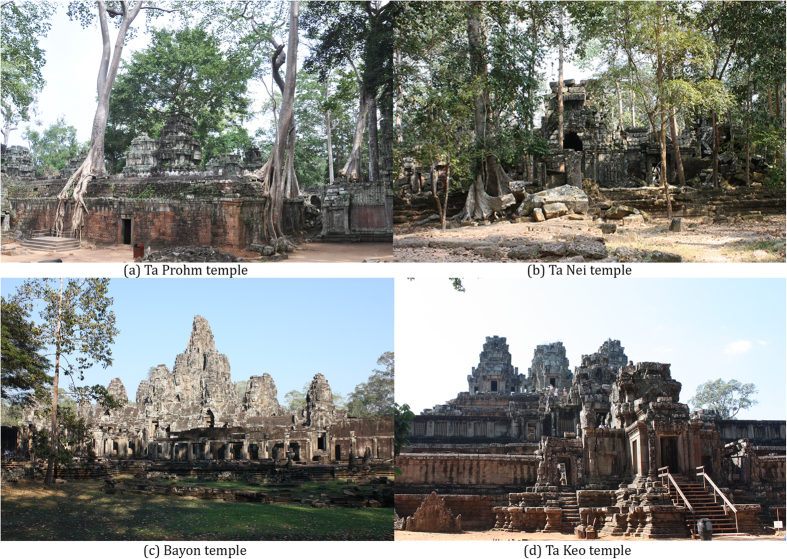
The four investigated temples in the Angkor archaeological area. (**a**) Ta Prohm; (**b**) Ta Nei; (**c**) Bayon; (**d**) Ta Keo. These temples are showed in an order that follows a gradient of increased humidity and light availability.

In the Angkor archaeological area, we selected four temples (Ta Prohm, Ta Nei, Bayon and Ta keo) to have a representative sample of different forest coverage (and hence of different microclimatic conditions) (Fig. 1). In particular, the Ta Prohm temple is surrounded by a dense forest canopy and, consequently, it shows conditions of high humidity and low light availability. The temple of Ta Nei presents a variable exposure to sunlight, with an external dense forest canopy, and an internal lower tree coverage. The Bayon temple is characterized by intermediate conditions of semi-shadow and moderate humidity. Finally, the Ta Keo temple presents sunny and dry conditions, due to the clearing of the forest occurred in the 1920 s.

We noted the environmental conditions (macroclimate and microclimate) of the archaeological site and of the examined temples. The macroclimate of the study area shows tropical monsoon characteristics. The area receives an average amount of rainfall of approximately 1,200 mm and is characterized by a wet season that starts in May and ends in November and a dry season from December to March^32^. Microclimatic data (Temperature, Light, and Relative Humidity) were collected for different monuments in the Angkor archaeological area^36^,^37^ and in case of the Ta Nei temple, they were analytically recorded and analyzed from 2001 to 2013 by the National Research Institute for Cultural Properties in Tokyo (NRICPT), both for the interior and the exterior enclosure of the temple^38^. The analyzed temples are built with sandstone usually classified as feldspathic arenite or micaceous arkosic sandstone, which is a soft, fine-grained sandstone weakly cemented by clay and calcite with a porosity of about 13–19%. Laterite was used as filler within walls, pavements and platforms^39^.

### Qualitative data collection and field assessments

In total, we carried out about 200 field observations (test areas). These test areas were approximately 30 cm × 30 cm using as a centroid the transition between two communities. We randomly selected the test areas keeping in mind the variety of environmental conditions across the analyzed temples. At each test area, we visually assessed light and water availability, using synthetic indicators, as usually carried out in vegetation surveys^40^,^41^. In particular, we distinguished seven different levels (Table 3) for both light and water availability.

For each temple, we identified the different colonizing communities and their BPs according to the classification used by Caneva *et al*.^32^ and Bartoli *et al*.^25^. Specifically, we categorized eight types/communities: A) *Trentepohlia* sp. community, a pioneer community creating a reddish biofilm; B) Cyanobacteria biofilms of *Scytonema* sp. and *Gloeocapsa* sp., forming black patinas; C) Cyanobacteria biofilm in combination with the lichen *Endocarpon* sp.; various communities of lichens dominated by: D) *Lepraria* sp., forming greenish and powdery crusts with diffuse margins; E) *Pyxine coralligera* Malme forming green-greyish crusts with irregular to radiating margins; F) *Cryptothecia subnidulans* Stirt. forming whitish crusts; mosses (G); and vascular plant communities (H). As regards the vascular plant communities we found both perennial herbaceous ferns (genera *Adiantum, Asplenium, Ceterach, Selaginella*), and flowering plants (herbaceous and woody). The most representative annual herbaceous flowering plant was *Peperomia pellucida* (L.) Kunth. This species has a shallow root apparatus and it is native to American and African tropical regions (http://www.ipni.org). Among the tree species, *Tetrameles nudiflora* R. Br. and *Ficus altissima* Blume are the most representative and potentially harmful species since their roots can severely damage the masonry structure of buildings.

For any colonizing community and related BPs identified on the stone surface of the temples, we took note of the environmental factors that could possibly affect them^32^. These factors include inclination (horizontal/vertical) and exposure (N, E, W, S) of colonized surfaces, and their distance from the ground [lower surfaces can potentially receive a higher amount of humidity (from rising damp) than upper parts]. In addition, we quantified the shadowing as a function of coverage of the forest canopy using four classes (5–25%; 25–50%; 50–75%; 75–100%). We also noted changes in environmental conditions at areas of transitions (ecotones) between two different communities.

To investigate the ecological structure for each colonizing community and related BPs observed in the field, we repeated the following procedure at each site:

(a) Identification of an area without any obvious biological growth (point 0);

(b) observation of the pioneer community in adjacent stone surfaces;

(c) identification of the differences in environmental factor/s between the point 0 and the adjacent area with colonization;

(d) observation of the transitions (ecotones) among communities, if present;

(e) observation of the environmental factor/s associated with that ecotone stage.

In this way, we identified the communities and related BPs, and the transitions among them. We noted all the potential differences in environmental changes for transitions found at each temple in a binary matrix. We performed at least 10 observations for each transition (if present). Observational data were then organized in a general scheme synthesizing how environmental factors affect the distribution of communities and related BPs along ecological gradients. An accurate photographic documentation complemented field notes and observations.

### Statistical analyses of field observations

**Figure 2:**
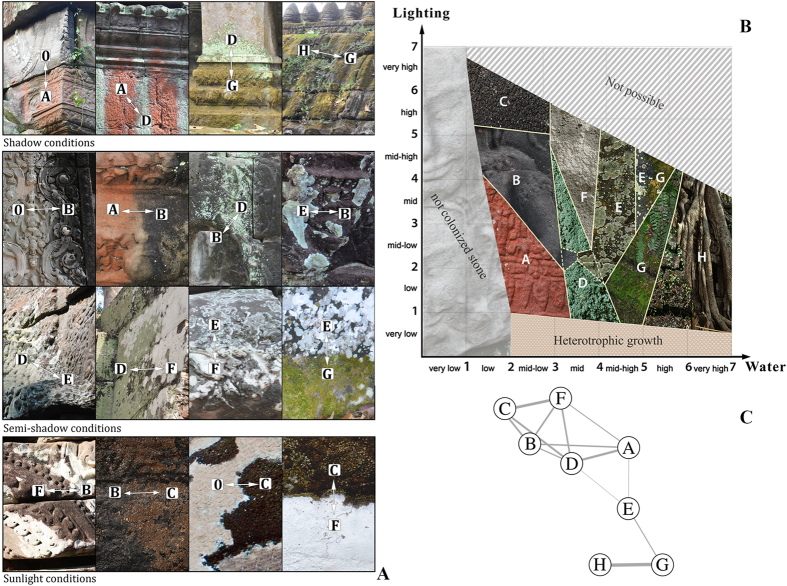
Ecological features of the eight colonizing communities (and related BPs) on the Angkor temples. (**A**) Observed transitions among the communities in various environmental conditions; (**B**) Ecological scheme showing the relations among the communities (and related BPs); (**C**) Network representing the transitions between different colonizing communities identified by the co-occurrence analysis. Line width is proportional to the association strength (“Weight” in Table 2) between two communities.

To confirm the potential transitions between colonizing communities, we assessed their degree of pairwise co-occurrence using the probabilistic approach introduced by Veech^14^. This method allows assessing, analytically, whether the overlap in the distribution of two taxonomic units (i.e., biological communities) is higher or lower than expected. In particular, Veech’s procedure can estimate the exact probability *P*(*j*) that two communities respectively occurring at *N1* and *N2* sites co-occur at exactly j sites (over a total of N sites available) using simple combinatorics. This probability *P*(*j*) is computed as the ratio between the number of ways in which the two units can co-occur at exactly *j* sites, and the total number of unique ways in which the two units can be arranged among all sites. Furthermore, this approach allows estimating the expected value of co-occurrence of two communities, i.e., to identify how many sites are expected to host both communities. Since the maximum possible value of co-occurrence is equal to the minimum of *N1* and *N2*, the expected co-occurrence value can be obtained by simply summing up *P*(*j*) × *j* from *j* = 0 to *j* = min (*N1*, *N2*). The difference between the expected and the observed co-occurrence value can then be standardized (i.e., divided by *N*) and used to compare co-occurrence between different pairs^14^,^15^. We used these values to build a network of associations (Fig. 2C) between the different communities.

To assess the relative importance of water and light availability in determining the ecological requirements of the colonizing communities and related BPs we used the statistical approach described in Strona *et al*.^35^. This method was originally conceived to identify the ranges of variability among closely related species for diagnostic purposes, however it can be applied to any case where a set of predictors consisting of value ranges (max-min) is associated to a categorical outcome variable. In the specific context of this paper, we used the following procedure:

- for each community, we generated 1000 simulated associations, each occurring at an hypothetical site having a value of light exposure randomly extracted from the range min (light) – max (light), and a value of humidity (as a proxy for water availability) randomly extracted from the range min (humidity) – max (humidity).

- We compared each one of these simulated associations with the light and humidity ranges of any (real) association, computing, for each simulated association, an “individual score” (*sc*) value^35^ as the number of times when both random values of light and humidity of the simulated association fell within the light and humidity ranges of a real association.

- After this procedure was replicated for each association, we computed an “overall score” (*SC*) value, as the total number of random values we generated, divided by the sum of all *sc* values. *SC* can vary between 1 (when each simulated occurrence has light and humidity values falling only within the light and humidity ranges of the association from which it originated) and 1/*T*, where *T* is the total number of considered associations (when each simulated association has light and humidity values falling within the corresponding ranges of all other associations).

- The same procedure was then performed using humidity and light ranges separately.

Finally, we compared the *SC* scores computed for the two measures separately and in combination. The larger is the *SC* value for a given factor (i.e., light and/or water availability), the higher is the importance of that factor for the survival of communities, and hence in determining their distribution. A high *SC* score indicates also the suitability of a factor for predictive models.

## Additional Information

**How to cite this article**: Caneva, G. *et al*. Combining Statistical Tools and Ecological Assessments in the Study of Biodeterioration Patterns of Stone Temples in Angkor (Cambodia). *Sci. Rep.* **6**, 32601; doi: 10.1038/srep32601 (2016).

## Figures and Tables

**Table 1:** Colonizing communities observed in the analyzed Angkor temples in relation to various environmental factors.

Inclination	Forest cover	Wateravailability	Very Low				Low				Med. Low				Med.				Med. High				High				Very High			
		Exposure	N	E	W	S	N	E	W	S	N	E	W	S	N	E	W	S	N	E	W	S	N	E	W	S	N	E	W	S
Vertical	0–25%		X	X	X	X	B	C	C	C	F	C	C	C	F	F	F	F	X	X	X	X	X	X	X	X	X	X	X	X
	5–25%		X	X	X	X	B	B	B	B	D	B	B	F	F	F	F	D	E	E	E	E	X	X	X	X	X	X	X	X
	25–50%		X	X	X	X	A	B	B	B	B	B	B	A	E	F	D	B	E	E	E	E	H	G	G	E	H	H	H	H
	50–75%		X	X	X	X	A	A	A	A	B	B	A	A	E	F	D	A	G	G	E	E	H	H	G	G	H	H	H	H
	75–100%		X	X	X	X	A	X	X	X	A	A	A	A	E	D	D	A	G	E	D	D	H	H	G	G	H	H	H	H
>Horizontal	0–25%		X	X	X	X	B-C	B-C	B-C	B-C	C	C	C	C	F	F	F	F	X	X	X	X	X	X	X	X	X	X	X	X
	5–25%		X	X	X	X	B-C	B-C	B-C	B-C	B	B	B	B	F-E	F-E	F-E	F-E	E-G	E-G	E-G	E-G	E-G	E-G	E-G	E-G	H	H	H	H
	25–50%		X	X	X	X	X	X	X	X	B	B	B	B	D	D	D	D	G-D	G-D	G-D	G-D	G-H	G-H	G-H	G-H	H	H	H	H
	50–75%		X	X	X	X	X	X	X	X	B	B	B	B	D	D	D	D	G-D	G-D	G-D	G-D	G-H	G-H	G-H	G-H	H	H	H	H
	75–100%		X	X	X	X	X	X	X	X	B	B	B	B	D	D	D	D	G-D	G-D	G-D	G-D	G-H	G-H	G-H	G-H	H	H	H	H

Colonizing communities with the corresponding dominant taxa: A = *Trentepohlia;* B = Cyanobacteria with *Scytonema* and *Gloeocapsa*; C = Cyanobacteria with *Endocarpon*; D = *Lepraria*; E = *Pyxine coralligera* Malme; F = *Cryptothecia subnidulans* Stirt.; G = Mosses; H = Higher plants; X = not detected.

**Table 2:** Results of the co-occurrence analysis aimed at identifying the transitions between two colonizing communities.

Co-occurrence analysis					
Transitions	ObservedTransitions	ObservedCo-occurrence	ExpectedCo-occurrence	p	Weight
***A-D***		19	11.083	0.000	0.417
***B-C***		9	4.500	0.000	0.500
***C-F***		9	4.500	0.000	0.500
***G-H***		10	3.611	0.000	0.639
***B-D***		24	15.167	0.000	0.340
***B-F***		19	10.833	0.000	0.408
***A-B***		17	10.292	0.000	0.353
***D-F***		18	11.667	0.000	0.317
C-D*	0	7	3.750	0.000	0.361
A-F*	0	11	7.917	0.016	0.162
***E-D***		21	19.333	0.029	0.069
***E-G***		12	10.472	0.029	0.118
A-E*	0	16	14.500	0.044	0.083
**B-E+**		19	17.722	0.064	0.058
E-H	0	9	8.056	0.079	0.094
**E-F+**		14	12.889	0.084	0.069
C-E	0	3	2.917	0.247	0.017
A-C	0	1	1.125	0.308	−0.042
C-G	0	0	0.833	0.682	−0.417
C-H	0	0	0.833	0.682	−0.417
F-H	0	2	4.444	0.929	−0.244
F-G	0	3	5.778	0.946	−0.214
D-H	0	4	6.667	0.955	−0.267
A-H	0	2	5.000	0.970	−0.300
A-G	0	3	6.500	0.982	−0.269
**D-G+**		5	8.667	0.990	−0.282
B-H	0	2	6.111	0.997	−0.411
B-G	0	3	7.944	0.999	−0.380

In italics the transitions identified by the probabilistic analysis. In bold, the transitions identified in the field; * = the transitions that were not identified by the qualitative ecological assessment in the field; += the transitions that were not identified by the statistical analysis). Observed transitions = the number of times the transitions were observed; Observed co-occurrence = the number of times the two communities listed in the column ‘Transitions’ were found in the same site; Expected co-occurrence = the number of times two communities were expected to be found together according to combinatorics; p = p-value that expresses the null expectation to find together the two communities a number of times equal or higher than the expected co-occurrence; Weight = interaction strength between the two communities, computed as: (Observed co-occurrence − Expected co-occurrence)/ min(*N1*, *N2*), with *N1* and *N2* being, respectively, the number of sites where the two target communities were found.

**Table 3:** Description of ranges for the synthetic indicators used to assess water and light.

Category (Light)	Range and description*
Very low	Indoor conditions without direct solar radiation
Low	Forest canopy 100–75%
Med. Low	Forest canopy 75–50%
Med.	Forest canopy 50–25%
Med. High	Forest canopy 25–5% or cleared areas in N exposure
High	Cleared areas in W-E exposures
Very high	Cleared areas in S exposure
**Category (Water)**	
Very Low	Vertical surfaces protected by incident rainfall in any exposure and in any condition of forest canopy cover
Low	Vertical and horizontal surfaces partially protected by incident rainfall in cleared areas in all exposures, and vertical surfaces with forest canopy cover between 50–75%
Med. Low	Vertical and horizontal surfaces exposed to incident rainfall, without percolation events - with forest canopy cover of 75–50% in S exposure; or with forest canopy cover of 50–25% in W-E exposures; or with forest canopy cover lower than 25% in N exposure
Med.	Vertical and horizontal surfaces exposed to incident rainfall, without percolation event - with forest canopy cover of 50–25% in S exposure; or with forest canopy cover lower than 25% in W-E exposures
Med. High	Vertical surfaces interested by percolation or rising damp (lower parts) with forest canopy cover lower than 25% in any exposure. Horizontal surfaces with forest canopy cover higher than 25%
High	Horizontal surfaces receiving incident rainfall, in semi shadow conditions with a forest canopy cover of 50–25%. Vertical surfaces interested by percolation or rising damp (lower parts) in any exposure
Very High	Horizontal surfaces receiving incident rainfall and interested by raising damp (lower parts), in shadow conditions under a forest canopy higher than 50%. Vertical surfaces interested by percolation or rising damp (lower parts) in any exposure

Notes: *Light: we considered a combination of these factors: cover of forest canopy (5 classes: 0–5%, 5–25%, 25–50%, 50–75%, 75–100%); exposure in cleared areas (3 classes: N, W and E, S). Water: we considered that water is inversely correlated to light (i.e., high solar radiation induces evaporation processes). We also considered forest canopy (5 classes: 0–5%, 5–25%, 25–50%, 50–75%, 75–100%); exposure of surfaces (3 classes: N, W and E, S); inclination (2 classes: vertical, horizontal); distance from the ground (higher parts, lower parts - interested with phenomena of rising damp); porosity of the stone (13–19% - see Uchida *et al*.^39^).
